# Molecular Survey on Kobuviruses in Domestic and Wild Ungulates From Northwestern Italian Alps

**DOI:** 10.3389/fvets.2021.679337

**Published:** 2021-06-14

**Authors:** Barbara Di Martino, Federica Di Profio, Serena Robetto, Paola Fruci, Vittorio Sarchese, Andrea Palombieri, Irene Melegari, Riccardo Orusa, Vito Martella, Fulvio Marsilio

**Affiliations:** ^1^Faculty of Veterinary Medicine, Università degli Studi di Teramo, Teramo, Italy; ^2^Istituto Zooprofilattico Sperimentale del Piemonte, Liguria e Valle d'Aosta, Centro di Referenza Nazionale per le Malattie degli Animali Selvatici (CeRMAS), Aosta, Italy; ^3^Department of Veterinary Medicine, Università Aldo Moro di Bari, Valenzano, Italy

**Keywords:** kobuvirus, wild boar, goat, chamoise, *Aichivirus B*, *Aichivirus C*

## Abstract

Since the first identification in 1989 in humans, kobuviruses (KoVs) have been identified from a wide range of animal species including carnivores, rodents, birds, ungulates, rabbits, and bats. Several studies have described the identification of genetically related KoVs in the fecal virome of domestic and wild animals suggesting a mutual exchange of viruses. By screening a total of 231 fecal samples from wild and domestic ungulates, KoVs RNA was detected in wild boars (3.2%; 2/63), chamois (4.6%; 2/43), and goats (2.6%; 2/77). On phylogenetic analysis of the partial RdRp sequence, the wild boar strains clustered within the species *Aichivirus C* whilst the strains identified in domestic and wild ruminants grouped into the species *Aichivirus B*. The complete VP1 gene was obtained for chamois and goat KoVs. Interestingly, upon phylogenetic analysis the strains grouped together with a KoV of ovine origin within a distinct genetic type (B3) of the species *Aichivirus B*.

## Introduction

Kobuviruses (KoVs) are small (~30–32 nm), icosahedral, non-enveloped viruses with a single stranded positive sense RNA genome of 8.2–8.4 kb in length, classified in the genus *Kobuvirus* within the family *Picornaviridae* (1). The viral RNA, polyadenylated at the 3′-end and covalently linked to a virus-encoded protein (VPg) at its 5′-end, consists of a 5′ untranslated region (UTR) of 646–717 nucleotides (nt), an open reading frame (ORF) of 7,308–7,467 nt and a 3′ UTR of 241–244 nt. The unique ORF encodes a single large polyprotein that is post-translationally cleaved into three distinct functional P regions (P1–P3) with P1 encoding the viral capsid proteins (VP0, VP3, and VP1) and P2 and P3 encoding proteins involved in protease processing and genome replication (2).

KoVs were first recognized in 1989 as the cause of oyster-associated non-bacterial gastroenteritis in humans in Aichi Prefecture, Japan (3). Since then, an increasing number of novel KoVs have been repeatedly identified from a large diversity of animal species, including ungulates, carnivores, rodents, birds, rabbits, and bats (4–21). Based on the phylogenetic analysis of the complete nt sequence of the VP1 encoding gene (15), the genus *Kobuvirus* is currently classified into six established species, *Aichivirus A* to *F*, and 20 genetic types [https://talk.ictvonline.org]. *Aichivirus A* includes a total of ten genetic types (*Aichivirus* A1–A10) identified in humans (A1) (3), canids (A2) (10, 11), rodents (A3, A6–A10) (14–17), domestic cats (A4) (12), and birds (A5) (18); *Aichivirus B* comprises KoVs detected in cattle (B1) (4), ferret (B2) (13), and sheep (B3) (6); within the *Aichivirus C* are classified KoVs detected in swine (C1) (5) and in goats (C2) (8); *Aichivirus D* includes newly discovered KoVs detected in Japanese black cattle (9), designed Kagovirus 1 and 2 (D1 and D2); the species *Aichivirus E* and *F* contains KoVs identified, respectively, in rabbits (E1) (19) and bats (F1 and F2) (21). Although information on the epidemiology of KoVs is still limited, cross transmission of these viruses has been revealed at the level of host order, family and species, with well-founded suspicious that wildlife ecosystem may constitute a large reservoir. This suggestion is supported by the recurrent identification of genetically related strains, practically indistinguishable from each other, in the fecal virome of domestic mammals and in their wildlife counterparts, such as *Aichivirus A-*2 (formerly canine kobuvirus) in dogs and wild canids (22–24), *Aichivirus C*-1 (formerly porcine kobuvirus) in pigs and wild boars (25, 26), *Aichivirus B*-1 (formerly bovine kobuvirus) and *Aichivirus C*-2 (formerly caprine kobuvirus) in domestic and wild ruminants (27). A molecular survey performed in the Serengeti National Park in Tanzania (23) has documented the circulation of highly genetically related strains between domestic and wild carnivores. In Serbia, porcine kobuvirus sequences identified from wild boars and domestic pigs revealed an amino acid diversity of 1%, emphasizing the role of wild boars as potential reservoirs for domestic pigs (26). In Italy, despite the identification of genetically similar bovine and caprine KoV strains in domestic ruminants and roe deer (27), systematic studies in a multi-host landscape exploring the role of different domestic and wild species in spreading and maintaining KoVs are still lacking. Herewith, we report the results of a molecular survey conducted in a defined geographical setting (Northwestern Italian Alps) where wild ungulates are abundant and occasionally enter in contact with livestock animals (seasonal grazing of goats and sheep).

## Materials and Methods

### Study Area and Sampling

**Figure 1:**
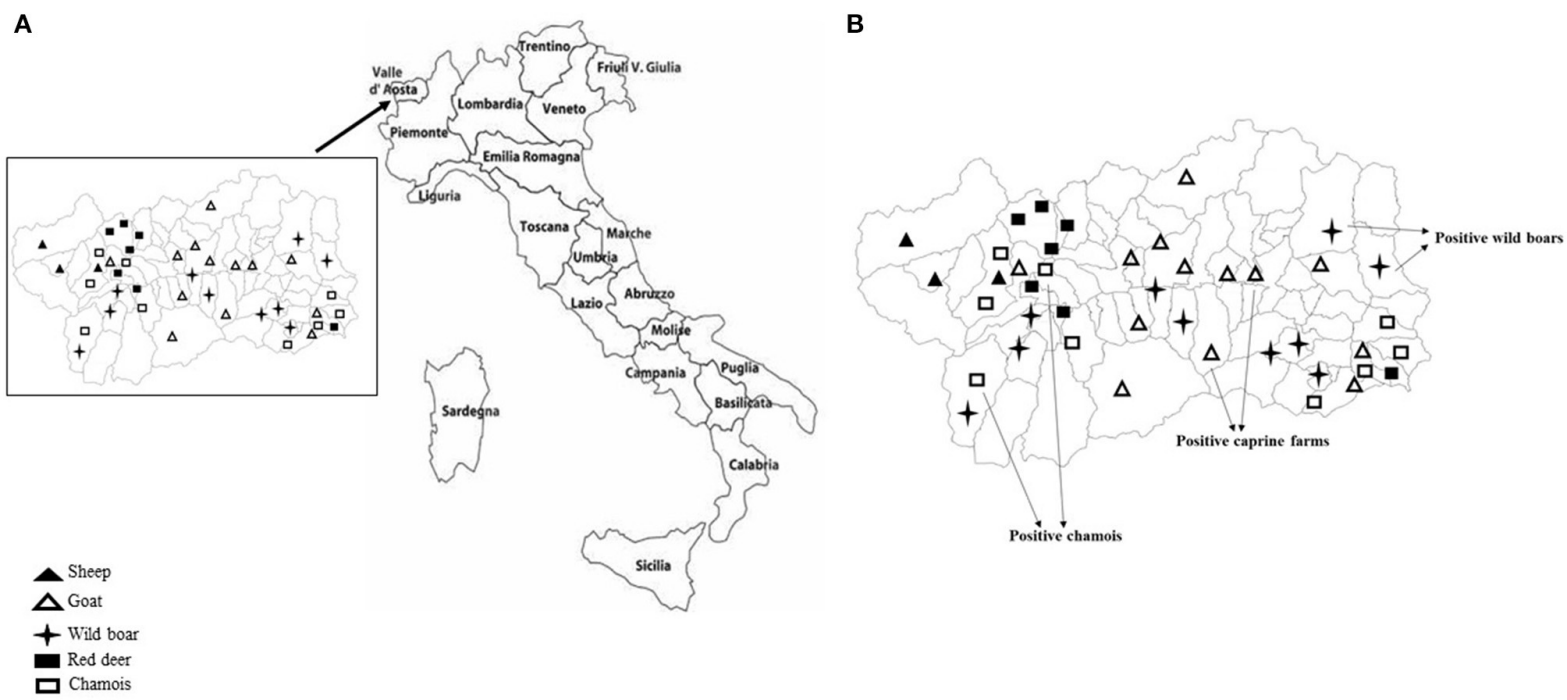
**(A)** Mapping of sheep and goat farms investigated and areas in which wild ungulates were sampled. **(B)** Sites where positive goats and wild ungulates were sampled.

Sampling covered the Region of Valle d'Aosta, in Northwestern Italy, with an overall geographic area of 3,261 km^2^. It is an alpine area characterized by the concomitant presence of domestic ruminant semi-rural farms and abundant ungulates wildlife. In this setting, the population of wild ungulates, composed primarily by wild boar (*Sus scrofa*), red deer (*Cervus elaphus*), and chamois (*Rupicapra rupicapra*), may occasionally enter in contact with domestic small ruminants through the use of pastures during seasonal transhumance or in the surrounding of backyard farms. Between September 2017 and December 2019, using a convenience sampling strategy, a total of 231 fecal samples was obtained from domestic and wild ungulates. Briefly, 95 fecal specimens were collected from 77 goats and 18 sheep, respectively, in 14 caprine and 3 ovine small farms from 16 municipalities in the Valle d'Aosta region (Figure 1A), consisting of 4–10 animals. All animals were female and clinically healthy at the time of sampling. On the basis of the age, 25 goats and 6 sheep were from the defined age group <3 years, 22 goats and 3 sheep from 3 to 4 years group, 16 goats and 9 sheep were from the age group >4 years. Age was unknown for 15 goats. Among wild animals, a total of 136 stool specimens was collected from 63 wild boars, 30 red deer, and 43 chamois sampled during the regular hunting season in Valle d'Aosta Region and submitted to the National Reference Center for Wild Animal Diseases (Italy). Age data were not available for many of the wild species investigated.

### Molecular Analyses

Total RNA was extracted from 200 μl of 10% (wt/vol) fecal suspension using TRIzol LS (Invitrogen, Ltd, Paisley, UK) procedure. KoVs RNA was detected by RT-PCR using broadly reactive primer pair, UNIV-kobu-F/UNIV-kobu-R (28) designed to amplify all members of the genus Kobuvirus and targeting a region of 217-bp of the viral RNA-dependent RNA polymerase complex (RdRp). In order to further investigate the genetic heterogeneity of the strains detected, all the samples yielding amplicons of the expected sizes were screened using specific primer sets (Table 1) designed by multiple alignment of sequences currently available in GenBank, able to amplify the complete VP1 encoding gene. All the positive amplicons were purified with the QIAquick Gel Extraction Kit (Qiagen, Milan, Italy) and sequenced by using BigDye Terminator Cycle chemistry (Applied Biosystems, Foster City, California, US). Basic Local Alignment Search Tool (BLAST; http://www.ncbi.nlm.nih.gov) and FASTA (http://www.ebi.ac.uk/fasta33) with default values were used to find homologous hits. Multiple alignments were performed using the commercially available Geneious software package version 9.1.6 (Geneious software package vers. 9, Biomatters, New Zealand, https://www.geneious.com/ Biomatters). Phylogenetic tree (neighbor joining and Kimura 2-parameter model) with bootstrap analysis (1,000 replicates) were constructed by using the MEGA software package, version X (29).

**Table 1:** List of primers used in this study.

**Oligonucleotide**	Position (nt)^*^	**Target gene**	**Sequence (5′ to 3′)**	**Sense**	**References**
UNIV-kobu-F	7493–7512	RdRp	TGGAYTACAAGRGTTTTGATGC	+	(28)
UNIV-kobu-R	7685–7707	RdRp	ATGTTGTTRATGATGGTGTTGA	−	(28)
2885-FW-est	2885–2904	VP1	TACACTGTGTGGGAYATCAA	+	This study
4388-REV-est	4388–4409	VP1	CAATGGATCTTGAGACACGGTG	−	This study
3794-FW-int	3794–3813	VP1	TTTGGCAACTTCCGTGGWTT	+	This study
4001-REV-int	4001–4020	VP1	TGGGTGTAGGTCATGCGCTG	−	This study
2803-FW-est	2803–2822	VP1	ATCTGGGTGATGAATCCACT	+	This study
4147-REV-est	4126–4147	VP1	GGTTTGCAGCCTGGACAACCTC	−	This study
3503-FW-int	3484–3503	VP1	GTGGTGAACACCACGTTTGG	+	This study
3773-REV-int	3754–3773	VP1	GTGTACGTCATGCGCTGGAC	−	This study

* *Nucleotide position refers to the sequence of the ovine kobuvirus prototype strain sheep/TB3/HUN/2009 (GenBank accession no. GU245693)*.

## Results

KoVs RNA was detected in a total of 6 fecal samples with an overall prevalence of 2.6% (6/231). Among the five ungulate species investigated in this study, viral RNA was found in wild boars (3.2%; 2/63), chamois (4.6%; 2/43), and goats (2.6%; 2/77), whilst all the samples collected from red deer and sheep resulted negative (Figure 1B). The two positive goats were aged 1 and 5 years old, respectively.

However, none significant association was found between the detection of KoVs RNA and age. Also, on the basis of the positivities obtained, a geographical clustering between wild and domestic animals was not observed.

**Figure 2:**
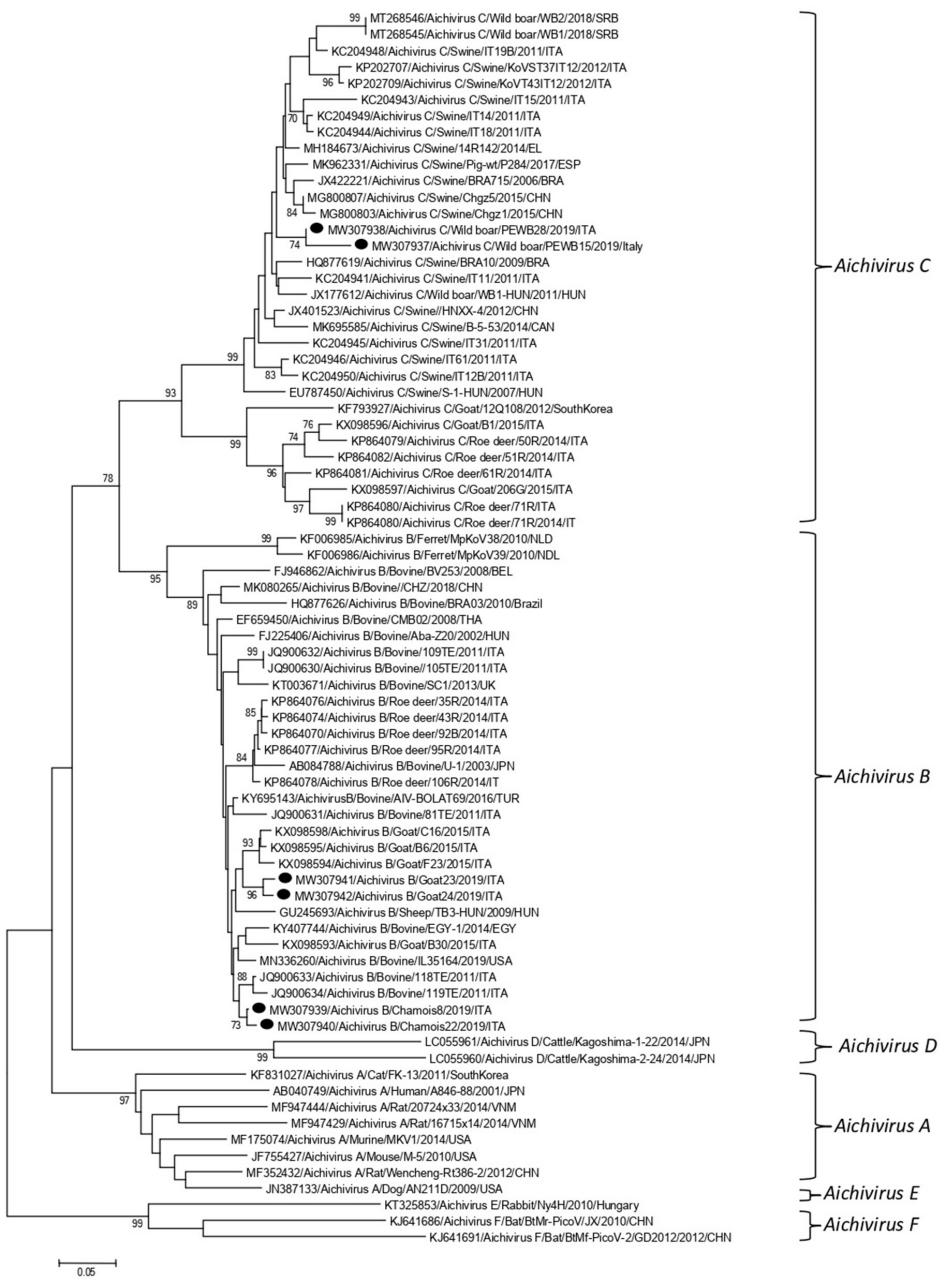
Phylogenetic tree based on the nt sequence of the partial RdRp gene of the KoV strains detected in this study. A selection of 70 sequences representative of the genus Kobuvirus was retrieved from GenBank. The tree was generated using the neighbor joining method and kimura 2-parameter model supplying statistical support with bootstrapping of 1,000 replicates. Labels indicate KoVs identified in this survey.

**Figure 3:**
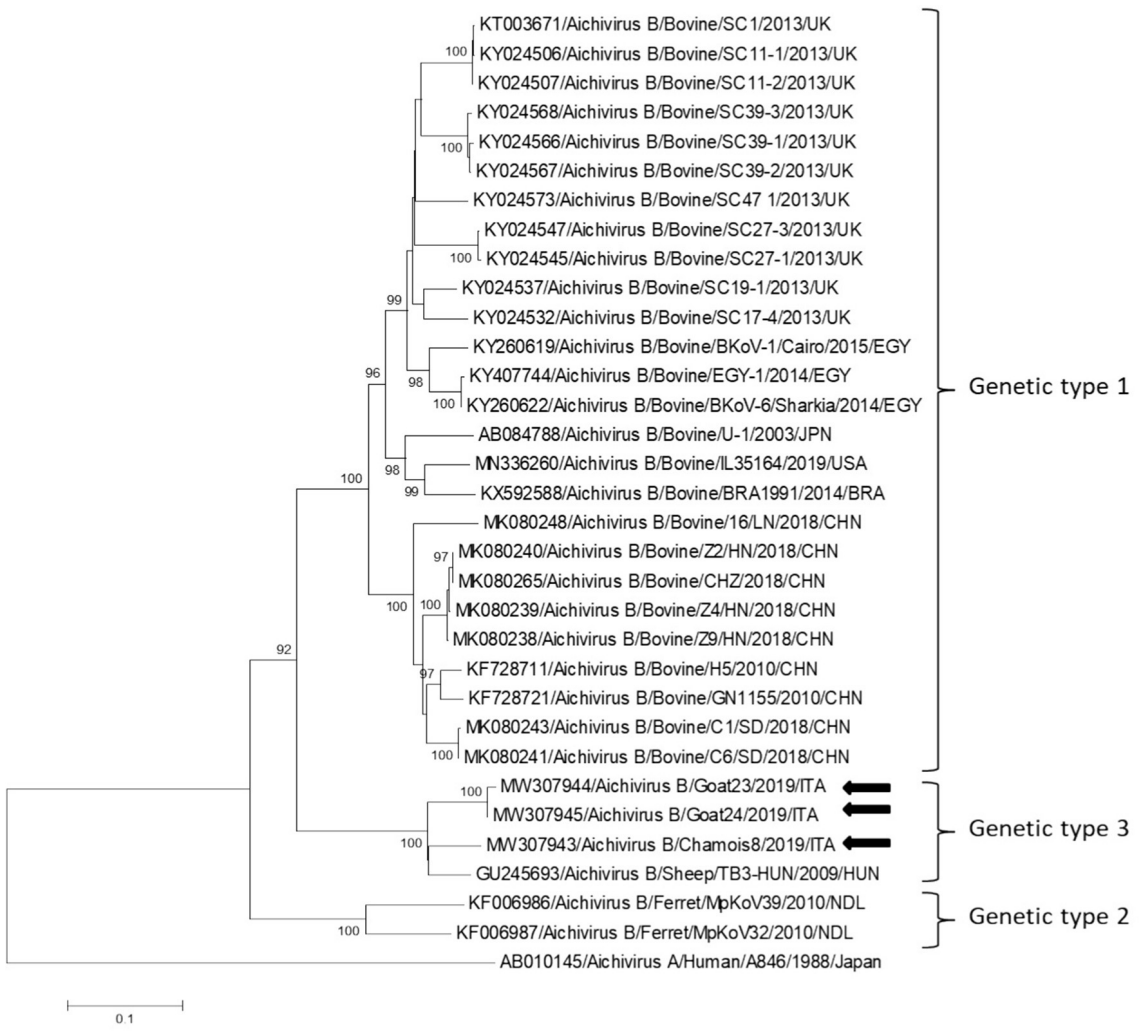
Phylogenetic tree based on the nt sequence of the VP1 encoding gene constructed with a selection of strains representative of the species Aichivirus B. Tree was generated using the neighbor-joining method with kimura 2-parameter model and supplying statistical support with bootstrapping of 1,000 replicates. The arrows indicate the KoV strains detected in this study. Human aichivirus A strain A8461 (GenBank accession no. AB010145) was used as outgroup.

Partial RdRp sequences were determined from the KoV positive samples. By sequence analysis, two wild boar strains, WB-15/ITA and WB-28/ITA (GenBank accession numbers: MW307937-8), shared 98.6% nt identity to each other and displayed a closed relatedness (94.0–96.1% nt identity) to porcine KoV sequences detected from swine enteric samples with or without diarrhea (30–35), whilst identities to KoVs identified in wild boar fecal samples (25, 26) ranged from 91.7 to 93.1%. On phylogenetic analysis, strains WB-15/ITA and WB-28/ITA clustered together with pigs and wild boar KoVs within the species *Aichivirus C* (Figure 2). Despite several attempts, additional genetic information was not obtained. The chamois KoV strains Chamois-8/ITA and Chamois-22/ITA (accessions MW307939-40) and the goat strains Goat-23/ITA and Goat-24/ITA (accessions MW307941-2) displayed the highest sequence identity (97.0–98.2% nt) to KoVs detected in calves and goats in previous surveys performed in an Italian study in Abruzzo, Southern Italy (36, 37). In the phylogenetic tree, the chamois, and goat KoV strains segregated within the species *Aichivirus B*, falling into two distinct clades. Strains Chamois-8/ITA and Chamois-22/ITA grouped with bovine KoVs, whilst strains Goat-23/ITA and Goat-24/ITA grouped with KoVs previously identified in goats and sheep (6, 37) (Figure 2). Out of the four KoV strains detected in ruminants, the complete VP1 encoding gene was obtained from three samples collected, respectively, from the two goats (Goat-23/ITA and Goat-24/ITA, MW307943-4) and a chamois (Chamois-8/ITA, MW307945). A selection of VP1 encoding gene representative of the *Aichivirus B* species was retrieved from GenBank. Based on the inspection of tree and according to the distance matrix, three genetic type groups could be distinguished (Figure 3). A large group included only viruses of bovine origin (*Aichivirus B*-1). The mean nt identity within this group was 88.7%. A second group, sharing more than 85.5% nt identity (mean identity 86.4%) comprised KoVs detected from ferrets (*Aichivirus B*-2). Group 3 included a strain of ovine origin, TB3/HUN/2009 (6), and the three KoVs found in goats and chamois in this study (*Aichivirus B*-3). Identity among these VP1 sequences was higher than 90% nt (mean identity 93.2%).

## Discussion

In this study we monitored circulation of KoVs among wild boars, goats and chamois in a restricted Italian geographical area. Identification of these viruses in wild boars and goats has been already described elsewhere (7, 8, 25, 26, 38, 39), whilst KoV infection has never been documented thus far in chamois. Viruses virtually identical to porcine KoVs (species *Aichivirus C*-1) were first identified in wild boars in Hungary (25) with a prevalence of 100% (10/10) and subsequently in Serbia with a rate of 6.0% (6/100) (26). Although the detection rate obtained in our survey was lower (3.2%, 2/63), our results indicate that circulation of porcine KoVs among wild boars is not uncommon and it is not limited to some settings (25, 26). In our investigation, by screening fecal samples from goat and chamois, only aichivirus B-like strains were identified, with the highest prevalence rate in chamois (4.6%, 2/43). Interestingly, all the four KoVs resulted genetically less related (92.1–95.5%) in the partial RdRp region to aichivirus B strains previously identified in roe deer from Valle d'Aosta Region (27) than to viruses detected in goats in Southern Italy (97.0–98.2%) (37). On analysis of the complete VP1 gene, a more definitive characterization of strains Goat-23/ITA, Goat-24/ITA, and Chamois-8/ITA was obtained, as all the strains grouped together with a KoV of ovine origin (TB3-HUN) in a separate genetic type (B3), distinct from viruses identified in calves (B1) and ferrets (B2). Accordingly, a definitive classification of KoV genetic types necessarily relies on sequence analysis of the full-length VP1 gene. The identification of KoVs in chamois, while expanding the host range of these viruses, reinforce the hypothesis that wildlife may represent an important reservoir of KoVs for livestock animals. However, despite the findings of this study confirmed a mild circulation of KoVs in wild and in domestic animals, a bi-directional flow was not revealed at least in the context analyzed. Further studies involving larger animal populations in other geographic areas where wild ungulates are in contact with domestics could be useful to obtain a more complete picture of the ecology of these viruses. In our analysis, all of the tested animals were apparently healthy at time of sampling. The role of KoVs in the etiology of enteritis in animals is still controversial (36, 40, 41). In previous studies (36), *Aichivirus B*-3 has been found at higher positivity rates in goats with enteritis (6.5%, 3/46) than in goats without enteritis (5.4%, 5/93), although the difference was not statistically significant. The possible role of *Aichivirus B*-1 as enteric pathogen involved in neonatal calf diarrhea has been hypothesed (41). Furthermore, in many cases, KoVs have been reported as the sole enteric pathogen detected in diarrheic pigs (42, 43), dogs (44), and cats (45, 46). Structured surveillance studies could help understand the overall impact of KoVs on livestock and wild animals in terms of health and production.

## Data Availability Statement

The data that supports the findings of this study are openly available in the GenBank database at https://www.ncbi.nlm.nih.gov/nucleotide/ under accession numbers MW307937-42 (BankIt2404995).

## Ethics Statement

For this study ethical statement was not required (Decreto Legislativo 4 March 2014, n. 26). Faecal samples from wild ruminants were collected during the routinely monitoring activities performed by the National Reference Centre for Wild Animal Diseases (CeRMAS – IZS PLV), whilst samples from sheep and goats were collected only for veterinary diagnostic purposes.

## Author Contributions

BDM, FDP, and SR: conceptualization. BDM, FDP, AP, PF, VS, and IM: methodology. AP, SR, PF, VS, FDP, IM, and RO: investigation. BDM, FDP, VM, and FM: writing—original draft preparation. BDM, SR, and RO: funding acquisition. All authors have read and agreed to the published version of the manuscript.

## Conflict of Interest

The authors declare that the research was conducted in the absence of any commercial or financial relationships that could be construed as a potential conflict of interest.
